# Soluble Receptor for Advanced Glycation End Products (sRAGE) is decreased in patients with Juvenile Idiopathic Arthritis (ERA category) and inversely correlates with disease activity and S100A12 levels

**DOI:** 10.1186/1546-0096-9-S1-P179

**Published:** 2011-09-14

**Authors:** Amita Aggarwal, Arpita Myles, Vishad Viswanath, Yogesh Preet Singh

## Objectives

Membrane bound receptor for advanced glycation end products (RAGE) is over-expressed in response to increasing concentrations of its ligand (eg S100A12) and triggers an inflammatory immune response. Its truncated form sRAGE acts as decoy receptor and competes for ligands thus down-modulating inflammation. Decreased sRAGE levels are associated with rheumatoid arthritis, Sjogren syndrome and Kawasaki disease, however limited data is available in JIA thus we studied its levels in JIA-ERA patients.

## Methods

sRAGE levels were estimated in serum of patients with ERA (n=101), SoJIA and polyJIA (n=10 each) and healthy controls (n=45). Synovial fluid (SF) sRAGE was measured in ERA, RA, ReA and OA patients (n=10). S100A12 levels were also measured. 24 ERA patients were followed up for 4 months. Disease activity was assessed by swollen joint count (SJC), tender joint count (TJC) and ESR.

## Results

Serum sRAGE (pg/ml) level was significantly lower in patients compared to healthy controls [515 (64-1887) vs 1542 (627-3159); p<0.0001]. In paired samples, SF had lower levels compared to corresponding plasma level [102 (51-799) vs 481 (134-1006); p<0.0001].

S100A12 (ng/ml) was higher in SF (1042; 573-1415) than sera (638; 208-779). Serum sRAGE negatively correlated with S100A12 levels (r= -0.474; p<0.01.), ESR (r= -0.306; p<0.01), SJC(r= -0.237; p<0.05) but not with TJC (r= -0.134; p=ns). The levels of sRAGE remained stable over time in patients with stable disease.

## Conclusion

sRAGE levels are reduced in patients with ERA and negatively correlate with disease activity and S100A12 levels. sRAGE may be a modulator of inflammation in these patients.

